# Differential miRNA signatures in Hepatitis E Virus Infection: Insights into acute, chronic, and pregnancy-related outcomes

**DOI:** 10.1007/s10096-025-05207-4

**Published:** 2025-07-14

**Authors:** Nancy León-Janampa, Julien Marlet

**Affiliations:** 1 INSERM U1259 MAVIVH, Tours University and Tours University Hospital, Tours, France; 2 Department of Bacteriology-Virology-Hygiene, Tours University Hospital, Tours, France

**Keywords:** MicroRNAs, Hepatitis E virus, Acute and chronic hepatitis, Liver failure, Clinical outcomes

## Abstract

**Purpose:**

Hepatitis E virus (HEV) is a positive single-stranded RNA virus that causes acute and chronic hepatitis with severe complications in pregnant women and immunocompromised individuals. HEV infection manifest asymptomatically or progress to fulminant hepatitis, liver failure, and extrahepatic manifestations such as neurological disorders and renal damage. MicroRNAs are non-coding RNA molecules that play a key role in diseases and viral pathogenesis, influencing viral replication and immune evasion through their interaction with host factors.

In this review, we discuss the role of microRNAs described to date in hepatitis E infection, highlighting their impact on acute, chronic, and pregnancy-related clinical outcomes.

**Results:**

miR-122, miR-214, miR-221, and miR-222 participate in the HEV replication and immune evasion, while specific miRNA profiles distinguish acute and chronic hepatitis E. HEV poses severe risks in pregnancy, with miR-431, miR-654, and miR-1468 related to self-limiting infection, and miR-450b to acute liver failure. Studying these miRNAs lead to biomarkers and therapies for hepatitis E.

## Introduction

The hepatitis E virus (HEV, *Paslahepevirus balayani*) is the leading cause of both epidemic and sporadic acute hepatitis in adults worldwide [1, 2]. Each year, around 20 million HEV infections occur globally, leading to 3.4 million symptomatic cases of hepatitis E and 44,000 fatalities [3, 4]. HEV currently impacts one-eighth of the global population, with a rising incidence in developed nations [5]. HEV is transmitted through the fecal–oral route or zoonotic transmission [6]. There are enveloped (sera) and non-enveloped (feces) HEV virions, suggesting different entry mechanisms [7]. The transmission from animals to humans represents a significant global public health concern, and HEV is ranked among the top 10 viruses with the highest risk and zoonotic transmission potential [8]. Porcine HEV has been identified in pigs in the United States, Canada, Spain, New Zealand, India, Japan, and China [9].

HEV infections have different clinical manifestations, ranging from asymptomatic cases to instances of fulminant or chronic hepatitis [10]. Typically HEV infection is acute and self-resolving, but it can become severe in pregnant women, particularly in the third trimester, resulting in a high occurrence of hemorrhage, acute liver failure, and elevated mortality rates (15%−30%) [1, 11–13], with an estimated 3,000 fetal deaths per year [14]. The molecular mechanisms underlying maternal mortality due to HEV remain elusive.

MicroRNAs (miRNAs or miRs) are non-coding single-stranded RNA molecules which regulate several human genes [15, 16]. Studies have demonstrated an association between miRNAs and various diseases, such as cancer, neurodegenerative diseases, cardiovascular diseases, diabetes, and viral infections [17–20]. Viruses genome contains only the essential components required for their propagation [21]. They regulate their transcription, translation, and post-translational modifications (e.g., glycosylation in some viruses) by balancing the efficient use of host proviral factors and defending against the host immune response [22]. Efficient viral replication requires the formation of an active viral replication complex, which is a tightly controlled process [22]. Viral pathogenesis is a complex event involving dynamic interactions with various host factors, among which miRNAs play a key role [23]. In the liver, miR-122 is predominant and contributes to liver homeostasis and viral replication [24]. In hepatitis C virus (HCV), miR-122 promotes replication by stabilizing the viral genome, while other host miRNAs (e.g., miR-448,  miR-let-7b) exert inhibitory effects [16, 25]. Hepatitis B virus (HBV) encodes two viral miRNAs (HBV-miR-2 and HBV-miR-3) that downregulate replication and be associated with oncogenesis [26]. Hepatitis A virus (HAV) is the first cytoplasmic RNA virus shown to encode miRNAs (hav-miR-1-5p, hav-miR-2-5p, hav-miR-N1-3p), which suppress replication and support persistent infection [27]. Several HEV-derived miRNAs, including the experimentally confirmed HEV-miR-A26 and nine additional candidates identified through in silico analysis, are proposed to contribute to viral replication and spread by interfering with host immune signaling and cellular pathways; however, further experimental evidence is needed to validate their expression and biological roles [28, 29]. Host miRNAs, notably miR-122 and miR-214, play a critical role in the HEV cycle by directly enhancing viral replication through binding to conserved genomic regions and modulating host immune and cellular responses [23, 30]. miR-122 facilitates replication and suppress interferon responses, while miR-214 promotes viral protein translation and thrombin-mediated replication processes [23, 30]. Additionally, HEV infection induces distinct changes in host miRNA expression profiles. Altered levels of several miRNAs (e.g., miR-122, miR-125b, miR-99a) have been associated with acute and chronic HEV infections [31–33]. In silico, several miRNAs (including miR-129–2-3p, miR-130a-3p, and miR-138-5p) have been identified through protein–protein interaction network analysis as being associated with chronic HEV infection in immunosuppressed patients, highlighting their potential role in disease progression and as targets for further validation [34].

This comprehensive review explores generalities of HEV in humans, the miRNA profiles described to date for acute and chronic infections and severe liver failure associated with pregnancy due to HEV infections. Furthermore, the possible roles of these miRNAs during HEV infection and the most interesting miRNAs proposed as diagnostic biomarkers are described.

## An introduction to HEV

Viral infections of the liver are among the most common infectious diseases worldwide and can be caused by several taxonomically unrelated viruses such as HAV, HBV, HCV and HEV [35–37]. HEV (genus: *Paslahepevirus*, family: *Hepeviridae*) comprises eight genotypes, where genotypes 1–4 are known to infect humans, with a reported case of genotype 7 infection as well [6]. HEV-1 and -2 spread through fecal–oral transmission, mainly via contaminated water, causing large-scale outbreaks and epidemics in developing countries [3], with a particular impact on pregnant women in their third trimester [13]. These genotypes are highly prevalent in low-income regions such as Asia and Africa [38]. In contrast, genotypes 3 and 4 are associated to zoonotic infections that are primarily endemic in pigs and represent an emerging public health concern in industrialized nations due to their widespread presence among humans and animal reservoirs [1, 38–40]. Although these genotypes can usually be resolved in immunocompetent individuals, but it can progress to chronic hepatitis E in immunocompromised individuals, mainly reported for HEV-3 and 4.

HEV infection presents a wide range of clinical manifestations, from asymptomatic cases to fulminant liver failure. It typically causes an acute, self-limiting hepatitis, with a prodromal phase lasting up to one week, featuring nonspecific symptoms such as fever, malaise, nausea. This is followed by jaundice, dark urine, and pale stools even prolonged cholestasis [41]. Although jaundice is reported in 60% of diagnosed cases, many infections go undetected [42]. Alanine aminotransferase (ALT), aspartate aminotransferase (AST), and bilirubin levels are usually elevated [43]. In immunocompetent individuals, HEV infections generally resolves spontaneously within 4 to 6 weeks and typically it remain asymptomatic or manifest as mild, self-resolving viral hepatitis without permanent liver damage [1]. However, individuals with underlying liver conditions experience severe complications, including acute liver failure [44, 45].

Detectable HEV RNA in a patient blood and persistent elevated liver enzyme levels for more than three months is indicative of chronic hepatitis E (CHE) [46]. Cases of chronic HEV infection have been reported in immunocompromised individuals, including solid organ (SOT) and hematopoietic stem cell transplant recipients, and those with human immunodeficiency virus (HIV) infection leading to adverse clinical outcomes [10, 47–49]. Also, chronic hepatitis E leads to liver diseases, including hepatic fibrosis, cirrhosis, acute decompensation, and liver failure [50, 51]. In a meta-analysis, the estimated pooled prevalence (95% CI) of HEV infection in patients with SOT was 20.2%, where the HEV prevalence for each organ transplant was: liver (27.2%), kidney (12.8%), heart (12.8%) and lung (5.6%) [52]. Therefore, patients with SOT have a considerable risk of HEV infection, but the clinical and virological factors associated with disease chronicity remain unclear [33].

In pregnant women, HEV infection begin with mild symptoms but can rapidly progress to acute liver failure, accompanied by serious complications such as cerebral edema, disseminated intravascular coagulation, and hepatic encephalopathy, which could result in death [40]. The infection is also associated with high rates of maternal and fetal mortality, especially in the third trimester, along with risks of preterm birth, low birth weight, and stillbirth [53].

HEV infections are not limited to the liver. Cases of HEV infection with extrahepatic manifestations have been reported, including neurological disorders, such as Guillain-Barré syndrome, inflammatory polyradiculopathy, neuralgic amyotrophy, bilateral brachial neuritis, myelitis, encephalitis, multifocal neuropathy and necrotizing myositis as well as hematological complications, pancreatitis and kidney damage [54–58]. The pathophysiological mechanisms by which HEV contributes to the development of extrahepatic symptoms have not been fully elucidated. One possible explanation could be that viral infections trigger a variety of host defense mechanisms that can cause cross-reactions between viral epitopes and autoantigens, leading to multisystemic manifestations [54]. Another possibility is the extrahepatic replication of HEV, according to evidence observed in experimental animals [59]. Also, HEV exhibits the ability to traverse physiological barriers such as the blood–brain barrier [60], the placental barrier [61], and the reproductive tract [62, 63], leading to infection of the central nervous system, fetus, and reproductive tissues, respectively.

Vaccination or treatment for hepatitis E is limited [64]. The only authorized vaccine (HEV239, Hecolin) has shown efficacy in healthy Chinese subjects, and several ongoing studies have focused on global acceptance [65, 66].

A post-hoc analysis of a phase 3 clinical trial conducted in China showed that the exposure to the Hecolin vaccine was not associated with an increased risk of fetal loss or neonatal abnormalities, supporting its safety in women of childbearing potential, including those who are pregnant or become pregnant [67]. Pharmacological therapy involves the nucleoside analog ribavirin, but it is often accompanied by clinical side effects [68]. Furthermore, previous studies have suggested that ribavirin promote the selection of viral quasispecies associated with treatment failure and viral recurrence [69–71]. Currently, the lack of effective treatments and the limited availability of vaccines underscore the importance of developing better prevention and management strategies to control hepatitis E globally.

## Molecular basis of HEV

HEV is a positive-sense single-stranded RNA virus with a genome length of approximately 7.2 kb [23]. The HEV genome contains a short untranslated 5′ region (5′UTR) with a 5′-m7G cap, three open reading frames (ORFs), and a short 3-UTR with a poly(A) tail that mimics mRNA after infection [72]. Non-structural proteins are encoded by ORF1 (~ 5.1 kb), whereas ORF2 (~ 2.0 kb) encodes the capsid structural protein, which plays a critical role in virion assembly and viral attachment to host cells [73, 74]. HEV ORF1 (pORF1) encode: methyltransferase (MetY), a fatty acid binding-like domain (FABD-like), a proline-rich (PRR) or hypervariable region (H), a macro-domain X (X), a helicase (HEL/NTPase) and RNA-dependent RNA polymerase (RdRp) (Table 1) [75].

**Table 1:** pORF1 organization in HEV-1 and HEV-3. Limits of HEV ORF1 domains from [75]

pORF1 domain	HEV-1 Sars55 strain	HEV-3 Kernow C1-p6 strain
MetY	1–506 aa	1–506 aa
FABD-like	515–707 aa	515–707 aa
PRR	ND	ND
macro-domain X	778–926 aa	850–998 aa
HEL/NTPase	930–1207 aa	1002–1279 aa
RdRp	1226–1693 aa	1298–1765 aa

ORF3 (~ 342 nt) encodes a small phosphoprotein involved in virion release [76, 77]. An additional fourth open reading frame (ORF4) within ORF1, is present in genotype 1 [78]. ORF4 (~ 478 nt), encodes a protein considered essential for the optimal function of RdRp in HEV-1 viruses [79]. ORF4 plays a crucial role in enhancing viral replication and promoting viral protein translation, contributing to the ability of the virus to cause severe infections [78].

The viral genome has limited coding capacity because of its small size; therefore, the virus relies heavily on host factors for energy, viral particle synthesis, stability, regulation, transport, and genome assembly [80]. To facilitate the entry, replication, and release of fully assembled virions, the virus employs several strategies: i) formation of a viral replication complex on intracellular membranes to protect viral RNA and protein components from degradation [81]; ii) the interaction between viral factors and essential antiviral pathway components to inhibit their function [82], and iii) reprogramming of host cell metabolism to exploit host factors and create a favorable environment for survival inside the host cell [83].

Viral components can be recognized by host pattern recognition receptors (PRRs) to induce antiviral immunity in the host [84]. Virus recognition involves a large family of related pattern receptors, such as Toll-like receptors (TLRs) and RIG-I-like receptors (RLRs). RLRs in the cytoplasm, such as RIG-I and MDA5, detect single-stranded HEV RNA. Binding of viral RNA to PRRs activates intracellular signaling pathways such as the NF-κB and IRF3/IRF7 pathways [85]. These pathways promote the production of type I interferons (IFN-α and IFN-β) and pro-inflammatory cytokines. Type I interferons are secreted and activate the interferon-stimulated gene system (ISGs), which degrades viral RNA, inhibits viral translation and replication, and enhances autophagy to clear viral complexes [86, 87]. HEV can partially evade the innate immune response via viral proteins, such as ORF3, which interferes with interferon signaling and PRR sensing, facilitating viral persistence. During the early stages of HEV infection, suppression of IFN-β through modulation of SIRP-α expression has also been reported [85, 88, 89].

Proteins involved in the host innate immune response can be regulated by miRNAs, which interact with messenger RNAs (mRNAs) [15]. miRNAs can regulate gene expression in eukaryotes through RNA interference [90]. These miRNAs bind to target mRNAs to degrade them or inhibit their translation, modulating biological processes such as cell growth, cell death, and immune response [90, 91]. In the viral context, miRNAs can have antiviral or proviral effects, and are being explored as indicators of disease progression and responses to treatments [92]. miRNAs such as miR-122 and miR-125b have been shown to be elevated in hepatitis B and C infections, playing roles in both viral replication and liver inflammation [16, 93, 94]. Thus, miRNAs play a key role in the activation of the innate immune response to HEV infection.

## Generalities of miRNA

### Host miRNAs

miRNAs have approximately 21 to 25 nucleotides in length, that are evolutionarily conserved and regulate gene expression in eukaryotes through the RNA interference pathway [29, 90]. The precursor miRNA (pre-miRNA) of 60 to 80 nucleotides (nt) is cleaved in the cytoplasm by the RNase III enzyme Dicer to produce the 21 to 23 nt-long miRNA [29]. miRNAs bind to target mRNAs and regulate their expression by degrading mRNA or reducing mRNA translation [95, 96]. The target sites of several miRNAs are sometimes located in a single gene, synergistically repressing mRNA expression [97, 98]. miRNAs exhibit pleiotropy, meaning that each miRNA can target multiple mRNAs (on average, approximately 100) and a single mRNA can be targeted by several miRNAs [91]. Thus, more than 60% of human genes are predicted to be regulated by endogenous miRNAs [15, 95]. As a result, miRNAs play a significant role in regulating a wide range of biological processes, such as cell growth and death, the development and differentiation of tissues, and immune system responses [99–103]. miRNAs are present both intracellularly and in circulation, where they are stable in body fluids such as plasma and serum, making them potential biomarkers [92].

A virus can successfully infect a host by crossing its defense mechanisms and effectively using host factors for replication [104]. Host-derived miRNAs can participate in virus-host interactions by exhibiting proviral or antiviral effects [93, 105, 106]. The ability of tissue-specific miRNAs to enter the bloodstream has created opportunities to use circulating miRNAs as noninvasive indicators of disease progression and treatment responses [107, 108]. Research has demonstrated that miRNAs have potential therapeutic applications against various viruses, including human immunodeficiency virus-1 (HIV-1), herpes simplex virus (HSV), HCV and influenza [109].

Studies have identified that certain miRNAs, such as miR-122, miR-99a, miR-125b, miR-720, miR-22, and miR-1275 are elevated in individuals infected with HBV or HCV [110]. Furthermore, higher levels of circulating miR-122-5p, miR-125b-5p, miR-192-5p, miR-193b-3p, and miR-194-5p have been observed in patients HBeAg-positive compared to HBeAg-negative patients [111]. miR-125b is associated with multiple aspects of viral replication, including HBV DNA, HBsAg, and HBeAg [110]. miR-22 and miR-1275 are independently correlated with serum γGTP levels, a liver enzyme commonly linked to alcoholic liver disease or biliary obstruction but which can also rise in severe viral hepatitis [110]. miR-122 indirectly inhibits HBV replication in HBV-infected individuals [106]. Jopling et al. (2008) demonstrated that the direct interaction between miR-122 and HCV RNA enhances HCV replication, rather than HCV RNA degradation [112, 113]. Elevated serum levels of miR-122 predict increased inflammation in patients with chronic HCV [114, 115]. Another study reported that HCV infection is associated with overexpression of miR-20a, miR-92a, miR-122, miR-885-5p, miR-134, miR-320c, and miR-483-5p [116–118]. Elevated levels of circulating miR-122 and miR-20a, which persist in chronic HCV infection, are associated with liver inflammation caused by HCV and the advancement of fibrosis, respectively. [16, 116, 117]. Therefore, miR-122 has been implicated in various liver pathophysiological processes [119].

miR-122 is the most abundant miRNA in the liver**,** which is highly expressed in hepatocytes and regulated by the nuclear transcription factors of hepatocytes [95, 120]. Acute and chronic liver damage due to hereditary disorders, infection, or intoxication leads to deregulation of miR-122 levels in the liver [93, 120]. Furthermore, when the pattern of circulating miRNAs was identified in patients with hepatocellular carcinoma, circulating levels of miR-122 were recognized as important predictors for the development of hepatocarcinome (HCC) [120–123]. In patients with drug-induced liver damage or cholestatic liver disease, as well as in patients with chronic HBV and HCV infections, higher levels of miR-122 were detected in the blood, correlating with the degree of necroinflammation, risk of disease progression, and treatment response [124–127].

### Viral miRNAs

Recently, miRNAs encoded by viruses have been described [128, 129]. The miRNA encoded by Epstein-Barr virus (EBV) was first discovered in 2004 [130]. Subsequently, an increasing number of viral miRNAs or miRNA-like molecules have been identified through sequencing and bioinformatic prediction. Both DNA and RNA viruses encode miRNAs that mediate viral replication and host immunity [26, 27, 130, 131]. An *in-silico* study has identified putative viral miRNAs encoded by HEV and their possible human mRNA targets. These viral miRNAs can affect host gene expression, the viral life cycle, and pathogenesis [29]. Targeting these viral miRNAs can disrupt the viral cycle and restore the host's original expression profile [29].

## Host miRNAs associated with HEV replication

Several miRNAs have been associated with HEV infection, either acute or chronic. Some of these have even been associated with specific context such as infection in pregnant women or complications (acute liver failure) (Table 2).

**Table 2:** miRNAs profiles in acute and chronic HEV infection, and in pregnant woman

miRNA	Function in the Cell	Function During HEV Infection	HEV Infection	References
miR-122	Regulation of lipid metabolism, proliferation, and differentiation of hepatocytes. Regulates the type I interferon response through the RIG-I/MAVS pathway	Enhances HEV replication by directly binding to the viral genome in the RdRp region of ORF1, stabilizing viral RNA. Positive correlation between ALT and miR-122	Acute and chronic	[30, 33, 132]
miR-140	Post-transcriptional regulation of genes, involved in cellular processes such as proliferation and differentiation	Essential for HEV replication by interacting with the binding site in the HEV genome and recruiting hnRNP K, facilitating replication	in vitro	[22]
miR-155	Regulation of immune response, inflammation, cell proliferation and differentiation	It likely regulates the inflammatory response to minimize excessive tissue damage during HEV infection	Acute*/ *in vitro	[100, 132]
miR-23a	Involved in cell proliferation, differentiation, apoptosis, immune response, and metabolism	Probably involved in modulating the immune response during HEV infection	Acute	[132]
miR-214	Regulation of cell differentiation, apoptosis, coagulation, and cancer. Modulates the NF-κB pathway, affecting the inflammatory response	Interaction directly with the viral genome to enhance HEV replication and HEV genome translation. Additionally, regulates the expression of proviral factors such as thrombin and suppresses antiviral factors like OAS2	ND	[23]
miR-221	Decrease the expression of cyclin-dependent kinase inhibitor 1B (p27^kip1^), affecting cell proliferation Regulates cell proliferation, apoptosis, and differentiation	Downregulation of miR-221 and 222 in ORF3-expressing HEK293 cells enhance p27^kip1^ expression Downregulation of miR-221 reduces type I interferon response, favouring viral replication	Acute and chronic	[132, 133]
miR-222-3p	Decrease the expression of p27^kip1^, affecting cell proliferation Associated with the OAS3 gene and TRAFD1 to modulate innate response	Downregulation of miR-221 and 222 in ORF3-expressing HEK293 cells enhance p27^kip1^ expression Regulates the expression of genes related to antiviral response and inflammation	ND	[133, 134]
miR-99a-5p	Regulates cell differentiation and apoptosis	Probably involved in the regulation of viral replication and immune response	Acute, Chronic	[33, 135]
miR-194	Regulates cell differentiation, immune response and maintains hepatic cell function	Potentially involved in HEV and HCV infection. Reduces type I interferon signaling, favoring viral replication. Suppresses IFN-β production through inhibition of the RIG-I/MAVS pathway	Acute, Chronic, HCV/HEV	[31, 132, 136–138]
miR-885	Involved in the regulation of cell cycle and apoptosis	Probably associated to liver damage and viral replication in HEV. miR885 is increased in HCC, cirrhosis and chronic hepatitis B	Acute, Chronic	[31, 116, 132, 139]
miR-30a	Regulates cell differentiation and immune response	Reduces type I interferon signaling, favoring viral replication	Acute, Chronic	[31, 132]
miR-223	Regulates cholesterol homeostasis	miR-223 and miR-27a modulates lipid metabolism favoring viral replication	Acute, Chronic	[132, 140]
miR-27a	Regulates lipid metabolism and inflammation	Negatively regulation of apoliprotein E gene (APOE), which influence in viral replication	Acute, Chronic	[132, 141, 142]
miR-335	Regulates inflammation and cellular signaling	Downregulation of miR-335 could lead to liver injury	Acute, Chronic	[31, 132, 143, 144]
miR-125b-5p	Involved in the regulation of cell proliferation, apoptosis, and immune response. Also act as tumor supressor in breast cancer	Probably involved in viral replication by modulating lipid metabolism	Acute, Chronic	[33, 33], [132, 134]
miR-192-5p	Involved in regulating metabolic pathways, fibrosis, and inflammation	Probably involved in the inflammatory response during HEV replication	Chronic	[33, 138]
miR-628	Involved in cell survival, growth, and inflammation	Changes in levels during HEV infection	Acute, HCV/HEV	[31]
miR-151-3p	Involved in cell proliferation, migration, apoptosis, and differentiation	Potentially involved in HEV and HCV infection and in the regulation of innate response	Acute, HCV/HEV	[31]
miR-526b	Regulator of proliferation, migration and invasion of tumor cells	Down-regulated in HEV infection	Acute, HCV/HEV	[31, 145]
miR-512-3p	Specific tumor suppressor in breast cancer	Potentially involved in HEV and HCV infection	Acute, HCV/HEV	[31, 146]
miR-1285	Regulator of cell proliferation and apoptosis	Changes in levels during HEV infection	Acute	[31]
miR-520b	Reduces the expression of multiple pluripotency regulators		Acute	[31, 147]
miR-302b	Involved in the regulation of pluripotency and cellular reprogramming		Acute	[31, 148]
miR-365a	Regulation of cell proliferation, metabolisme, innate immune response and apoptosis. Target genes MAPK1	Associated with acute liver failure in pregnant women, probably associated to apoptosis	ALF	[31, 32]
miR-365b			ALF	[32]
miR-188	Regulation of cell proliferation and apoptosis		ALF	[32]
miR-190b	Participates in the regulation of activation and proliferation of CD4 + T cells		ALF	[32, 149]
miR-374c	Regulation of cell proliferation and apoptosis. Upregulated in mice with ALF-induced hepatic encephalopathy		ALF	[32]
miR-450a-1	Regulation of cell proliferation and apoptosis		ALF	[32]
miR-450a-2	Regulation of cell proliferation, apoptosis, cell migration and invasion		ALF	[32, 150]
miR-450b	Regulation of cell proliferation, apoptosis, and inflammatory processes. Target gene RNF20	Strong predictor of acute liver failure and death in pregnant women with HEV	ALF	[32]
miR-504	Regulation of cell proliferation and apoptosis	Regulates cell proliferation and apoptosis in HEV-infected pregnant women	ALF	[32]
miR-580			ALF	[32]
miR-616			ALF	[32]
miR-2115			ALF	[32]
miR-3117			ALF	[32]
miR-4482			ALF	[32]
miR-4772			ALF	[32]
miR-5690			ALF	[32]
miR-590	Regulates antiviral response by targeting the IL-6 receptor		acute, ALF	[32]
miR-561	Regulation of cell death and DNA metabolic processes		acute, ALF	[32]
miR-624			acute, ALF	[32]
miR-651	Regulation of cell proliferation and apoptosis		acute, ALF	[32]
miR-877			acute, ALF	[32]
miR-627	ND	Related to HEV infection in HEV-infected pregnant women	acute, ALF	[32]
miR-3143	ND		acute, ALF	[32]
miR-3605	ND		acute, ALF	[32]
miR-3656	ND		acute, ALF	[32]
miR-3940	ND		acute, ALF	[32]
miR-5189	ND		acute, ALF	[32]
miR-431	Regulation of cell regeneration and apoptosis	Associated with spontaneous resolution of HEV infection in HEV-infected pregnant women	Self-limiting acute	[32]
miR-654			Self-limiting acute	[32]
miR-1468	Recruitment of neutrophils and resolution of pathogen damage		Self-limiting acute	[32]
miR-4435	ND		Self-limiting acute	[32]

*ND* not data, *ALF* Acute liver failure

Among these miRNA, mir-140 has been extensively described in the context of HEV infection by Patil et al. (2023). This mir-140 binds HEV RNA and a specific RNA-binding protein (RBP, hnRNP K) [22]. RNA-binding proteins (RBPs) and miRNAs are two major classes of molecules involved in the regulation of gene expression [22]. The interaction of miRNAs or RBPs with cis-acting elements is essential for the pan-genotypic replication of HEV [151]. Cis-acting elements in HEV RNA are specific sequences or structures that allow interaction with cellular proteins or molecules, facilitating viral replication. RBPs is able to interact with specific RNA sequences, patterns, or structures [152–154]. Host RBPs influence the permissiveness of specific cell types and pathophysiology of viral infections [22]. Heterogeneous nuclear ribonucleoprotein K (hnRNP K) is a highly conserved and abundantly expressed RBP. hnRNP K be involved in various functions of RNA virus replication, such as RNA processing, splicing, transcription, and translation [155–157]. During HEV replication, hnRNP K has been shown to interacts with RNA-dependent RNA polymerase [22, 158].

The miR-140-hnRNP K interaction has been previously reported in the replication of HCV [21]. In HEV, the viral genome contains an miR-140 binding site (MBS) in cis within ORF1 [22]. The cis-acting elements of HEV act as scaffolds, attracting both viral and host factors to assemble the viral replication complex [22]. The secondary structure of MBS can serve as an assembly site for the viral replication complex [151]. For efficient HEV replication, the host factor miR-140 establishes a platform at the conserved MBS, and hnRNP K can only identify the HEV MBS in the presence of miR-140, allowing the recruitment of other proteins to the HEV replication complex [22]. This replication complex include Ago2-hnRNPK-miR-140. In addition, the secondary structure of the HEV genome play a significant role in its translation [151].

Other host miRNAs, such as miR-214, miR-122, miR-221, and miR-222 have also been reported to play a role in HEV replication [23, 30, 133]. miR-221 and miR-222 are negatively regulated by swine HEV ORF3 in the human embryonic kidney cell line 293 [133]. In silico, candidate target genes of miR-221 and miR-222 were found to participate in the expression of cyclin-dependent kinase inhibitor 1 B,  (p27^kip1^) [133]. p27^kip1^ is a cell cycle regulator and tumor suppressor. Down-regulation of p27^kip1^ can lead the increased hepatocyte proliferation contributing to liver fibrosis and HCC development [159]. It is presumed that the negative regulation of miR-221/miR-222 induced by swine HEV ORF3 enhances the expression of p27^kip1^ in HEK293 cells [133].

miR-214 directly interacts with HEV RNA to enhance HEV replication and genome translation [23]. Overall, miR-214 plays an important regulatory role in chondrogenesis, bone development, and embryonic development [23, 160, 161]. It is also a central player in various types of cancers, including HCC, melanoma, myeloma, and glioma, as well as in acute kidney injury and cardiac hypertrophy [162–166]. It has been observed that miR-214 levels increase during liver injury and are involved in viral infections [23, 167]. In silico studies have shown that the binding sites for in the viral genome are significantly conserved among HEV genotypes and related RNA viruses [22, 23].

Similar to miR-140, miR-214 directly interacts with the highly conserved binding site of miR-214 in the HEV genome (ORF1) [23]. This interaction fine-tunes the expression of host factors and creates a supportive viral environment. Increased translation results in elevated levels of HEV ORF2, which is responsible for the positive regulation of miR-214 [23]. This synergistic effect resulted in elevated levels of miR-214 and HEV RNA. It has been previously demonstrated that viral RNA acts as a sponge for miRNA, attracting cellular miRNAs within infected cells [23, 168, 169]. This lead to competition between viral RNA and host mRNAs for binding endogenous miRNAs [23]. During the early stages of the viral cycle, endogenous miR-214 be attracted to viral RNA rather than to host transcripts [23]. However, HEV ORF2 induces sufficient miR-214 expression to balance the action of miR-214 on HEV RNA as well as on host transcripts [23]. Therefore, miR-214 is considered a key host factor for HEV replication [23].

**Fig. 1:**
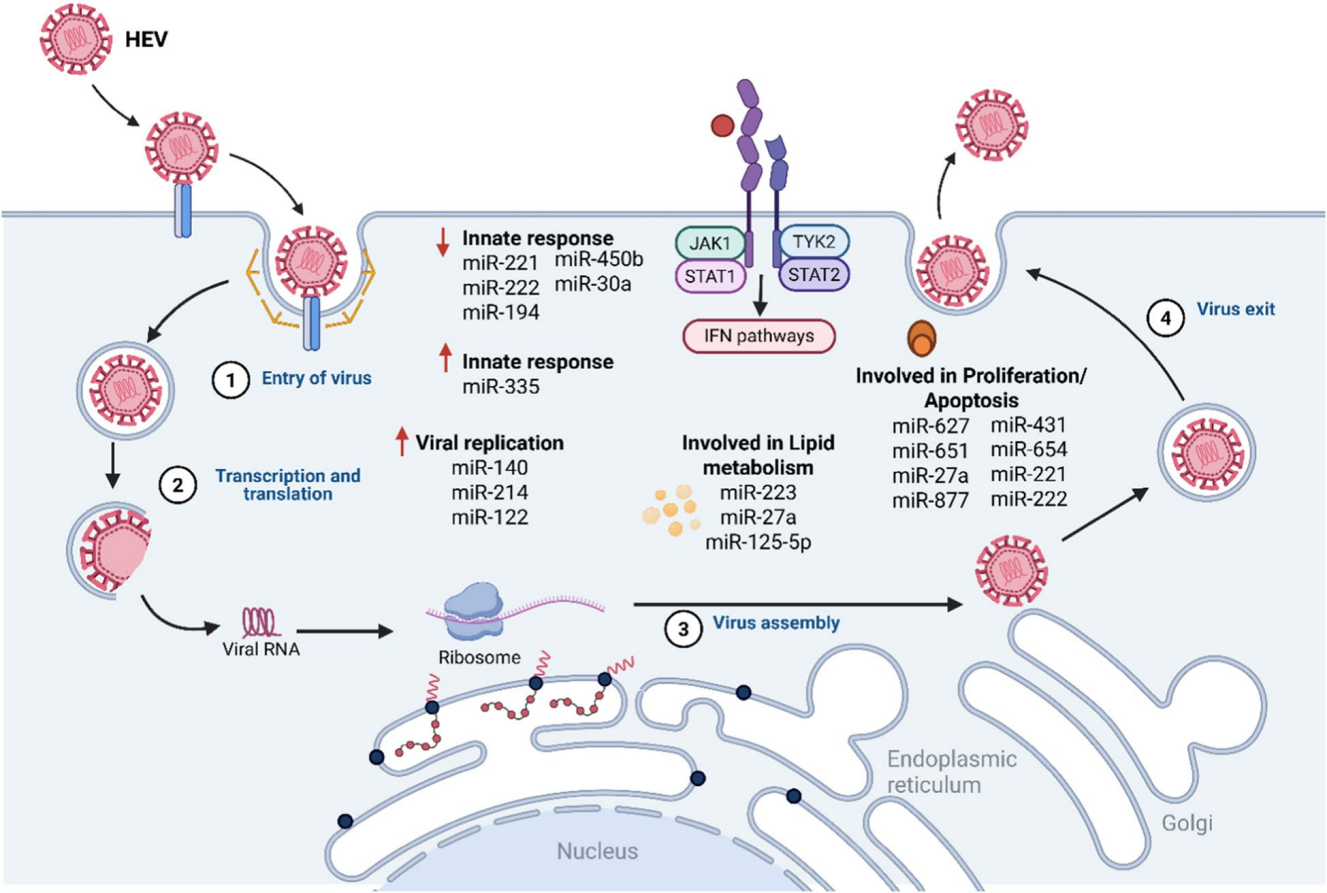
Modulation of miRNAs profiles in HEV-infected cells. During HEV infection different host miRNAs are able to regulate different pathways into the cells and participate in viral replication. Red arrows indicate the activation (up) or inhibition (down) of the processes indicated in the figure. Image prepared using BioRender

miR-122 is a crucial regulator of hepatitis virus replication [30]. Overall, miR-122 plays a central role in liver development, homeostasis and differentiation [119]. HEV has a positive-sense RNA genome and initiates the synthesis of viral proteins immediately after entering the host cells [30]. In silico, it was shown that most genomes (203/222) from different genotypes of HEV (1–4) harbor at least one binding site for miR-122 [30]. The HEV-1 genome contains a highly conserved binding site for miR-122 (97%) in the RNA-dependent RNA polymerase (RdRp) region [30]. Mutations in the RdRp of HEV-1, specifically at the miR-122 binding site, resulted in the complete inhibition or significant reduction of HEV replication [30]. This suggests that regulating miR-122 levels could aid in treating HEV infection in humans [30]. An inhibitor of miR-122, miravirsen (SPC3649), has successfully undergone Phase II clinical trials for HCV [170], and could also be employed for the treatment and management of hepatitis E. In vitro, exogenous overexpression of miR-122 facilitated HEV-1 replication in different cell lines S10-3 and HepG2/C3A [30]. This indicates that miR-122 facilitates HEV-1 replication, likely through the positive regulation of HEV ORF1 by miR-122 [30] (Fig. 1).

## Host miRNA in acute and chronic hepatitis E infection

The mechanisms underlying the diverse clinical outcomes of HEV infections remain poorly understood. However, it has been observed that specific miRNA profiles during viral infection reflect the cellular processes associated with viral replication and disease progression [132]. In acute hepatitis E, miR-122, miR-221, and miR-222 are implicated in the regulation of HEV [133, 171].

Previous study reported that 51 potential miRNAs from plasma could be regulated by HEV, indicating that HEV infection alters the expression or secretion of these miRNAs [132]. Samples of patients with symptomatic acute hepatitis E (AHE) and blood donors (BD) without prior treatment were considered for this analysis. No differences were observed between exposed and untreated BDs, suggesting that no particular deregulation persisted after the clearance of HEV infection [132]. Acute infection with HEV-3 was associated with the positive regulation of miR-122, miR-194, miR-885, and miR-30a, and with the negative regulation of miR-221, miR-223, and miR-27a, compared to untreated BDs [132]. Patients with AHE exhibit significantly higher levels of miR-122 and miR-194 and lower levels of miR-221, miR-27a, and miR-335 than BDs with HEV [132]. Decreased expression of miR-221 in HEV-infected groups [132] has also been observed during HCV infections [31, 172]. This distinct miRNA pattern in acute hepatitis E enhance viral replication and reduce antiviral immune responses. miR-194, miR-335, and miR-221 can discriminate between asymptomatic HEV infections and those that develop acute symptoms. miR-335 is able to discriminate patients with symptomatic acute hepatitis E and patients with chronic hepatitis E [132], suggesting that the diverse outcomes of HEV infection could result from different miRNA deregulations induced by HEV [132].

Elevated levels of miR-122 and miR-885 are associated with liver damage. Alterations in circulating levels of miR-122 have been associated with various liver conditions, including HCC, non-alcoholic steatohepatitis, and infections caused by HCV and HEV [33, 116, 173, 174]. Likewise, elevated levels of extracellular miR-885 have been observed in patients with liver diseases such as cirrhosis, HCC, acute HCV infection, and chronic hepatitis B [31, 116, 139]. However, increased circulating levels of miR-122 and miR-885 in all HEV-infected groups, compared to untreated BDs, are observed even in the absence of apparent liver damage. So, HEV triggers the upregulation of these miRNAs [132].

miR-122 did not show statistical significance as a diagnostic marker to differentiate between asymptomatic HEV infections and those developing clinical symptoms, but its positive correlation with ALT levels suggests it could act as a biomarker for liver injury during HEV infection [132]. These findings align with earlier studies that established a direct correlation between miR-122 levels and the clinical presentation of viral hepatitis [132]. For example, circulating levels of miR-122 were associated with severe hepatitis E during pregnancy [171] and correlated with alanine aminotransferase levels during HBV, HCV, and chronic HEV infections [33, 116, 127]. Furthermore, miR-122 has been demonstrated to play a critical role in HCV replication by stabilizing the viral genome, which enhances the replication process [112, 175]. Therefore, the positive regulation of miR-122 in all HEV-infected groups could be part of the mechanism by which HEV promotes its replication, which is consistent with the positive correlation observed between miR-122 and plasma viral load [132].

The upregulation of miR-30a and miR-194 in various viral infections has been associated to the modulation of antiviral immune responses, particularly through the negative regulation of type I interferon signaling [136, 176]. Interestingly, plasma exosomes from patients infected with HCV or co-infected with HCV and HEV showed elevated levels of miR-194, a miRNA highly expressed in hepatocytes [137]. This suggests that miR-194 expression be altered in response to infections with both HCV and HEV [31]. Circulating miR-194 positively regulated in HEV-infected groups is consistent to reduce type I IFN production [132].

miR-223 and miR-27a play roles in regulating cholesterol homeostasis and lipid metabolism, respectively [140, 142]. The repression of miR-27a has been associated to increased cellular lipid levels and enhanced HCV replication [142]. Furthermore, miR-27a negatively regulates the expression of multiple genes associated with lipid metabolism, such as apolipoprotein E (APOE), which could influence HEV replication [141]. The elevated levels of miR-27a observed in HEV-infected groups represent a mechanism through which HEV promotes viral replication by altering lipid metabolism pathways [132].

Moreover, miR-335 has been shown to play a critical role in activating inflammatory signaling pathways [143, 144]. The circulating levels of miR-335 demonstrated high accuracy and reliability in differentiating between patients with asymptomatic HEV infection and those with AHE [132]. This suggests that reduced levels of miR-335 during HEV infection could contribute to liver damage [132]. Additionally, earlier research revealed that exosomal levels of miR-335 were disrupted in both HEV and HCV infections [31], with a notable downregulation observed in symptomatic HEV patients but not in asymptomatic cases. Plasma levels of miR-335, miR-194, and miR-221 are able to discriminate between asymptomatic and symptomatic HEV infected patients, even differentiate acute infections from those progressing to a chronic stage [132].

Symptomatic infection by HEV in patients with acute hepatitis E was associated to the downregulation of miR-335, potentially contributing to liver inflammation. Additionally, it was associated with increased disruption of lipid metabolism via miR-223 and miR-27a, as well as greater impairment of type I IFN signaling through miR-194, miR-30a, and miR-221[132]. Additionally, circulating levels of miR-335, miR-194, and miR-221 have potential as predictive markers for HEV infection outcomes [132]. So, HEV infection modifies the host miRNA expression profile involved in lipid metabolism and type I IFN response, suggesting that HEV replication could regulation of both processes [132].

In silico analysis in chronic HEV infection in kidney transplant patients showed that HEV-associated miRNAs and transcription factors were related to interferonsignaling pathways [134]. In these patients, Tumor necrosis factor receptor-associated factor-type zinc finger domain containing 1 (TRAFD1) is a key regulator to control excessive innate immune responses and control HEV infection [134, 177], by activation of the RNase L activation pathways, in association with the oligoadenylate synthetase (OAS) family proteins (OAS1, OAS2, and OAS3) [178]. miR-222-3p and miR-125b-5p interact with the IFN-induced gene OAS3 and TRAFD1 [134]. Thus, it is interesting to hypothesize that HEV could interact with miR-222-3p and miR-125b-5p to modulate the immune response during viral infection.

Also miR-222-3p is implicated in acute HEV infection [133]. In viral hepatitis, miR-222 is a potential biomarker in the diagnosis of liver lesions or progression, cirrhosis, and HCC [179, 180]. In acute and chronic HEV infection, miR-125b-5p act as a biomarker for early detection and differentiation between acute and chronic infection [33].

Harms et al. (2020) reported the initial profiling of 180 serum miRNAs and identified liver-specific miRNAs (miR-99a-5p, miR-122-5p, miR-125b-5p, and miR-192-5p) as potentially regulated in HEV infection [33]**.** Specific miRNA combinations, miR-99a-5p, miR-122-5p, and miR-125b-5p for acute hepatitis E with viremia (AHEv), and miR-99a-5p, miR-122-5p, miR-125b-5p, and miR-192-5p for chronic hepatitis E with viremia (CHEv), can distinguish acute from chronic infections compared to HEV-negative controls [33]. No association has been found between HEV-3 subtypes and disease severity [33]. Immunosuppressive drugs have no documented effects on hepatic miRNA expression, allowing valid comparisons between acute and chronic hepatitis E cases.

The expression levels of miR-125b-5p and miR-99a-5p were significantly reduced, whereas miR-122-5p and miR-192-5p were increased in CHE, with or without viremia, compared to AHEv, indicating their altered expression during chronic infection [33]. However, further research is needed to clarify whether transplant patients show lower miR-99a-5p and miR-125b-5p levels and higher miR-192-5p levels at HEV infection onset or during the transition from acute to chronic infection, and to explore miRNA profiles across acute and chronic phases in the same patients [33]. The roles of these miRNAs (miR-99a-5p, miR-122-5p, miR-125b-5p, and miR-192-5p) in HEV infection remain unclear, although they are known to maintain hepatic differentiation, suppress cell proliferation and apoptosis, and potentially support HEV replication [24, 135, 138, 181, 182].

Notably, miR-122-5p stabilizes the HCV genome, enhancing replication, and a similar role has been suggested for miR-122-3p in HEV. Additionally, the increased level of miR-122-5p in CHE patients with viremia compared to HEV-negative controls correlated significantly with ALT/AST levels, suggesting its potential as a biomarker for liver injury, although larger patient cohorts are required for validation [33]**.** Similarly, miR-122 levels are elevated in acute liver failure patients, regardless of the cause, suggesting their utility in reflecting massive liver cell death during acute viral infections [183]. These findings highlight the potential of miRNAs as noninvasive biomarkers for diagnosing and monitoring HEV infections, although further research is needed to confirm these mechanisms and refine their clinical applications.

Additionally, McGowan et al. (2020) reported miRNAs profiles between HCV and HEV analyzing miRNAs from exosomes of patients [31]. Exosomes play a significant role in liver disease, particularly in infections caused by HCV and HEV [31]. HEV has exosome-like envelope complicating their classification as enveloped or non-enveloped [184]. Research on miRNAs in HCV infection surpasses that on HEV, and no data exist on exosomes from HCV/HEV-co-infected patients due to their rarity. In HCV-related liver disease, miR-885-5p and miR-365 are upregulated, whereas miR-627-5p and miR-221 are downregulated [31]. In HEV infection, miR-526b is notably downregulated, and six miRNAs (miR-628-3p, miR-194, miR-151-3p, miR-512-3p, miR-335, and miR-590) could be involved in both infections [31]. Preliminary data from an HCV/HEV co-infected patient revealed 77 upregulated and 43 downregulated miRNAs after antiviral treatment [31]. These findings highlight the crucial role of miRNAs in liver disease caused by HCV and HEV co-infection. Differential regulation of specific miRNAs in these infections suggests potential biomarkers and therapeutic targets, emphasizing the need for further research, particularly in co-infected patients.

## Host miRNAs associated with HEV infection in pregnant women

Infection with the HEV is typically a mild and self-limiting condition, but it can become life-threatening in individuals with pre-existing liver disease or during pregnancy [185]. Approximately 25% of pregnant women infected with HEV experience acute liver failure, leading to significant risks for both the mother and fetus, including high rates of illness and death [186]. Mechanisms associated to rapid progression toward liver failure in pregnant women with HEV infection remains unclear. Liver failure could be related with decreased activation of protective miRNAs, which are typically present in self-limiting HEV infections [32]. This failure result in the destruction of functional liver cells and subsequent liver failure [32, 187].

In normal pregnancy, a reduction of more than 50% in miRNAs and genes is observed in pregnant women, compared non-pregnant women [32]. This could be explained by immunosuppression or non-functional immune cells during pregnancy [188, 189]. The miRNAs in the pregnant group preferentially targeted the gene expression profiles of neutrophils, macrophages, monocytes, NK cells, B cells, T cells, plasmacytoid DCs, and eosinophils [32]. Also significant changes occur in the coagulation system [23]. Overall, the mechanisms associated with HEV infections in pregnant women be due to the interaction of various miRNAs in specific immune pathways, leading to an inflammatory response, liver failure, or death [32].

HEV infection can affects the coagulation system during pregnancy, leading to substantial deregulation [186]. This HEV-induced coagulopathy be responsible for the more severe infection during pregnancy, although the mechanism remains unclear [23]. Thrombin is a crucial player in the coagulation system and primarily synthesized in the liver. Thrombin is rapidly produced during liver injury [190]. Deregulation of the coagulation system plays a central role in the pathogenesis of HEV [23, 186], especially in fulminant hepatitis E [191]. Reports indicated that patients with hematological neoplasms have a high prevalence of HEV infection [23, 58]. So, the activity of intracellular thrombin is a critical factor necessary for HEV replication [23].

miR-214 and HEV ORF3 increase intracellular active thrombin, which is a host proviral factor for HEV replication [23, 192]. miR-214 represses the expression of the negative regulator of thrombin, protein C (PROC) [23]. Interaction of miR-214 with HEV RNA showed positive regulation, whereas the interaction of miR-214 with cellular mRNA transcripts of PROC and OAS2 showed conventional negative regulation [23]. OAS-2 is a member of the OAS family that primarily blocks viral infections [193]. The negative regulation of PROC enhances the expression of the proviral factor intracellular active thrombin [23], and negative regulation of OAS-2 minimize the innate antiviral response exerted by the host.

**Fig. 2:**
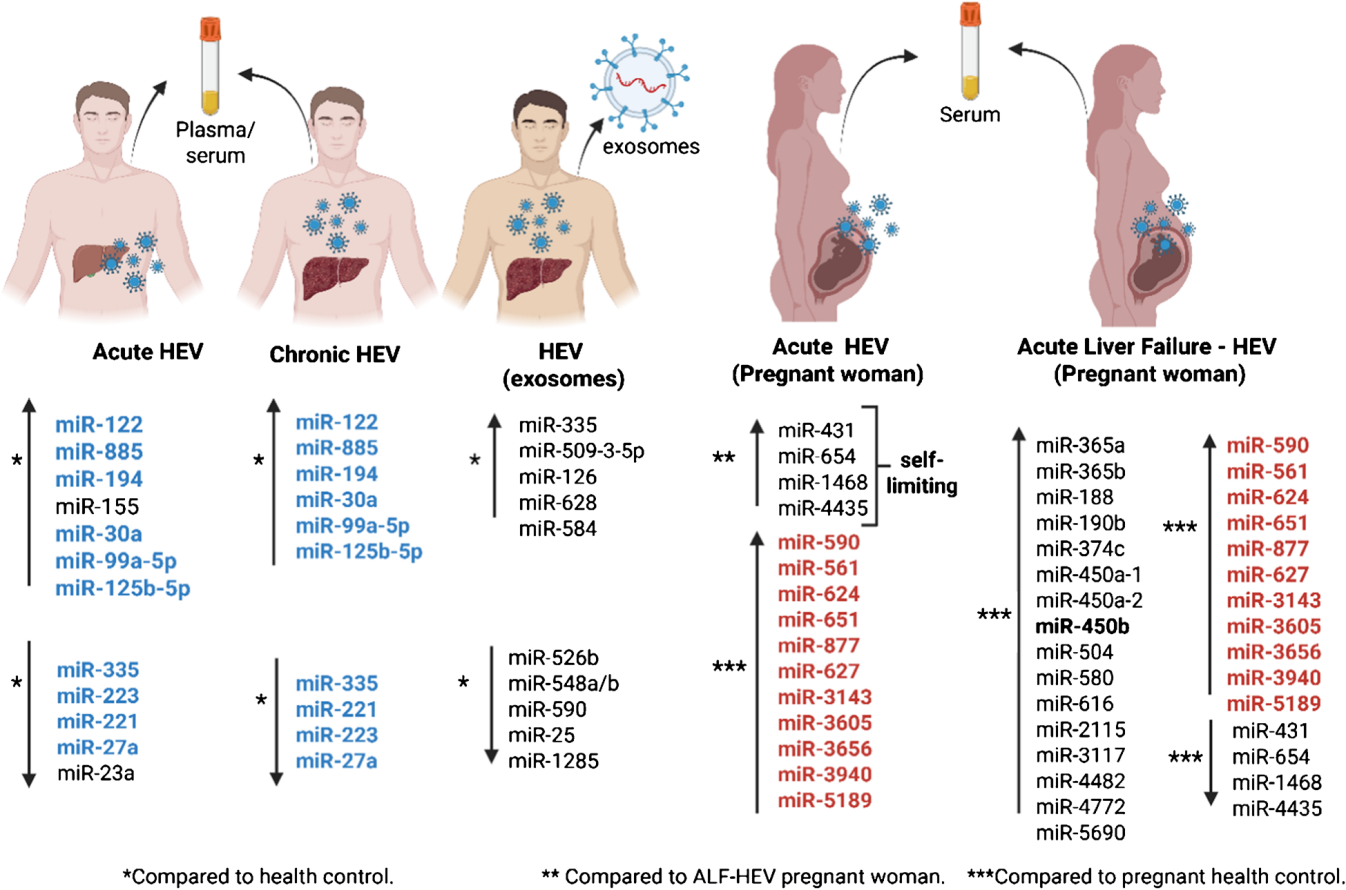
miRNA profiles in acute and chronic HEV infection, as well as in pregnant women with either acute HEV or HEV-induced acute liver failure. miRNAs with similar expression between acute and chronic HEV are highlighted in blue. In pregnant women with acute HEV or HEV-induced acute liver failure, similar miRNAs highlighted in red were compared to the expression of healthy control pregnant women. Up arrows indicate overexpression and down arrows indicate downexpression of miRNAs. The miRNA profile of acute, chronic and exosomes from HEV infected-patients were compared to health control. miRNA profile of acute and ALF from HEV infected-pregnant woman were compared to ALF-HEV and health pregnant woman, respectively. Image created using BioRender

Trehanpati et al. (2017) reported distinctive miRNA profiles between pregnant and non-pregnant women with self-limiting acute HEV infection or acute liver failure (ALF) [32]. Eleven miRs (miR-561, miR-590, miR-624, miR-627, miR-651, miR-877, miR-3143, miR-3605, miR-3656, miR-3940, and miR-5189) were specific to HEV infection during pregnancy (acute-HEV and ALF-HEV), compared to non-infected pregnant women [32]. These miRNAs are directly associated with cell death and DNA metabolic processes [32] (Fig. 2).

**Fig. 3:**
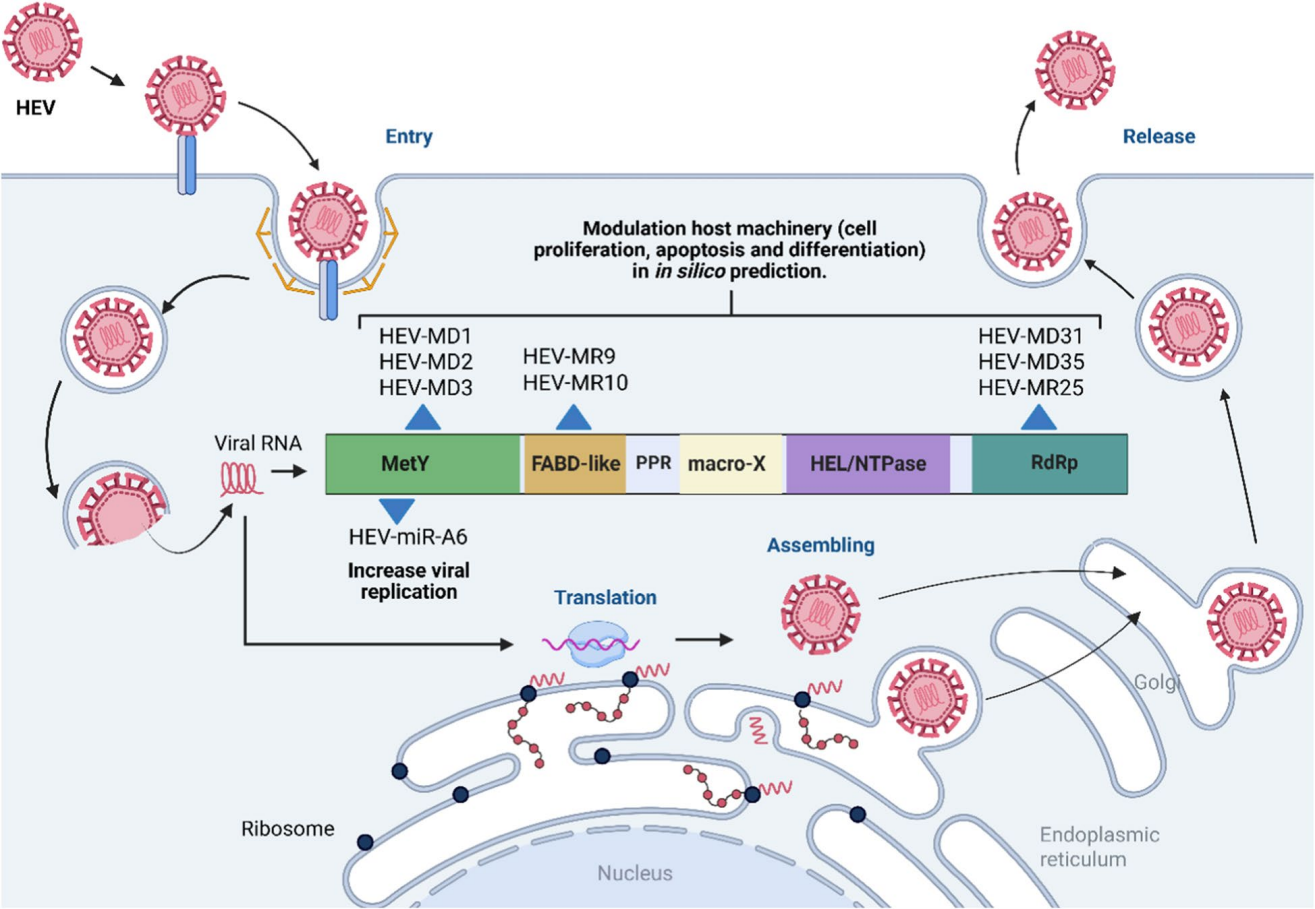
Distribution and functions of HEV pre-miRNAs and HEV-miR-A6 into HEV-1, considering the model pORF1 HEV-1 (Sars55 strain) recently described [75]. Image created using BioRender

miR-431, miR-654, miR-1468, and miR-4435 were clearly expressed in pregnant women with self-limiting acute HEV and healthy women, but reduced in patients with ALF [32]. Differential expression of these miRNAs in acute HEV, compared to ALF, was increased, however compared to health pregnant control, it was decreased (Fig. 3). So far, miR-431 and miR-654 have been reported in cell regeneration [194], proliferation, and cancer cell apoptosis [195], while miR-1468 is involved in resolving pathogenic assaults by recruiting neutrophils [196]. Therefore, these miRNAs have a protective effect on the self-cleansing of HEV infection.

In addition, 16 miRNAs (> two-fold change, FC) were overexpressed in acute liver failure HEV (miR-188, miR-190b, miR-374c, miR-365a, miR-365b, miR-450a-1, miR-450a-2, miR-450b, miR-504, miR-580, miR-616, miR-2115, miR-3117, miR-4482, miR-4772 and miR-5690) compared to self-limiting acute HEV and health pregnant women [32]. miR-365a-3p, miR-450a1-5p, and miR-450b-5p were validated in a large group of pregnant women with acute HEV, acute liver failure HEV, and healthy controls [32].

Among these, the dominant positive regulation of miR-450b in pregnant patients with acute liver failure HEV could regulate several genes involved in cellular inflammation and apoptosis [32, 197]. Therefore, miR-450b, induced by HEV, negatively impacts cell survival by promoting apoptotic cell death, sustained inflammation, and tissue damage, ultimately contributing to liver failure [32].

## HEV miRNAs and viral replication

During HEV replication, a negative-sense RNA intermediate is transcribed from positive-sense genomic RNA [29, 198]. This negative-sense RNA serves as a template for producing more positive-sense genomic RNA as well as subgenomic RNA [198]. The possible existence of these subgenomic RNAs makes HEV a suitable candidate for studying virally encoded miRNAs. Viral-encoded miRNAs regulate the viral cycle and evade the host innate immune system [28].

Through computational prediction modeling, nine possible HEV-related pre-miRNAs were identified. HEV-MD1 (97 nt), HEV-MD2 (95 nt), HEV-MD3 (52 nt), HEV-MD31 (76 nt), HEV-MD35 (89 nt), HEV-MD39 (85 nt), HEV-MR9 (97 nt), HEV-MR10 (56 nt) and HEV-MR25 (64 nt) [29, 199]. These HEV miRNAs could modulate host machinery during cell proliferation, apoptosis and differentiation. In silico*,* genes associated with metalloprotease ADAMTS-16, nitrogen-sulfur metabolism, vitamin B12 conversion and genes associated with immune response have been identified as targets of HEV miRNAs. Interestingly, high levels of ADAMTS-3 and ADAMTS-16 were reported in patients with recurrent pregnancy loss [200]. Therefore, the regulation of these markers by HEV-miRNAs could play a role in HEV-1 pathogenesis during pregnancy-associated abortions (Fig. 3).

HEV-miR-A6 (MetY, HEV ORF1) is an HEV-miRNA which is highly conserved among the eight genotypes of HEV. HEV-miR-A6 facilitate HEV replication and is detectable in patients and animal models with acute HEV infection [28]. Interestingly, HEV-miR-A6 was specifically detected in the liver, kidney, spleen and colon of HEV-infected BALB/c nude mice via in situ hybridization [28]. The expression of HEV-miR-A6 increases during HEV replication and is significantly higher in patients with acute hepatitis E than in those recovering [28].

HEV-miR-A6 overexpression modulates the innate immune response. Regulatory signaling protein α (SIRP-α) is an important glycoprotein involved in the negative regulation of innate immunity [88]. HEV infection activates SIRP-α to negatively regulate type I interferon β (IFN-β) [89]. Overexpression of HEV-miR-A6 activates SIRP-α to promote viral replication by inhibiting IFN-β [28]. In vivo, significant suppression of IFN-β production was observed in the serum of HEV-infected mice pretreated with HEV-miR-A6 [28].

## Conclusions

Several miRNAs, such as miR-122, miR-214, miR-221, and miR-222, have been shown to interact with the HEV genome, facilitating replication and evading the immune response. Furthermore, specific miRNA profiles have been observed in patients with acute and chronic hepatitis E, suggesting that these miRNAs could serve as biomarkers for diagnosing and monitoring infection. HEV infection can be particularly severe in pregnant women, with a high risk of liver failure and maternal and fetal mortality. Specific miRNAs, such as miR-431, miR-654, and miR-1468, have been associated with self-limiting infection, while miR-450b was associated with acute liver failure. This miRNA (miR-450b) be involved in regulating immune response and inflammation during pregnancy. In summary, miRNAs play a fundamental role in the pathogenesis of HEV infection by influencing viral replication, immune responses, and clinical outcomes. Their study offers promising prospects for the development of biomarkers and therapeutic strategies to control hepatitis E.

## Data Availability

No datasets were generated or analysed during the current study.
